# Rupture of the Urethra

**Published:** 1876-05

**Authors:** J. B. Hamilton

**Affiliations:** Assistant Surgeon U.S.A., Fort Colville, Washington Territory


### RUPTURE OF THE URETHRA.

By J. B. HAMILTON, Assistant Surgeon, U. S. A., Fort Colville, Washington Territory, September 15, 1875.

In the foreign selections of the London *Medical Times and Gazette* for June 19, 1875, an article appears from the *Gazette Hebdomadaire* of June 4th, wherein is reported a discussion by the Societe de Chirurgie upon the following question:

"What conduct should the surgeon pursue in cases of violent contusion of the perinæum, with rupture of the urethra, without external wound, but complicated with retention of urine?"

This question was illustrated by the recital of several cases, mainly treated by perineal incision.

I take the liberty of sending the notes of a case of rupture of the urethra, which was treated with ice bags to the perinæum, the aspirator being used for the relief of the retention of urine. This case recovered speedily, and with so little trouble as to leave nothing to be desired, and, as far as a single case can, it goes a long way toward decisively answering the question above propounded.

The aspirator used was of Tiemann's manufacture, and was similar to the one figured on page 538 of the London *Medical Times and Gazette*, May 15, 1875.

CASE.—*August* 20, 1874.—Was called at 10 A. M. to see Mike Cronin, aged nineteen years, an Irishman of spare habit, quite tall and thin. On arrival, found that he had been hauling wood, and the team becoming frightened by a stick failing on them, ran away. He was thrown upon the ground just in front of the fore wheel of the wagon. He fell upon his back, with his legs abducted; the wheel struck forcibly the perinæum and passed over the left groin. Assistance being near at hand, he was carried to the house of a farmer for whom he was at work, and was visited by me within an hour and a half.

On carefully examining him, there was no serious injury perceptible, but he complained of much pain in the groin and hip. One-half grain of sulphate of morphia was administered.

*August* 21*st*, 9 A. M.—Visited patient, and found him suffering very great pain from retention of urine; the bladder was considerably distended, and on inquiry it was found that no urine had been passed since the receipt of the injury, and some blood had escaped from the penis. The introduction of a No. 7 catheter was attempted, but when the point of the catheter arrived at the membranous portion of the urethra it passed no further into the urethral canal, but slipped easily out into the connective tissue of the perinæum on either side. The point of the catheter was quite obtuse, and could not have caused the laceration, as no force was used in its introduction. Copious hæmorrhage followed the withdrawal of the catheter. A second dose of sulphate of morphia was administered, and the patient was removed to his home two miles and a half distant, and an ice bag applied to the perinæum. At one o'clock Dr. P. Fenity, of Kane, Illinois, met me in consultation, and it was agreed to open the bladder by perineal section. While we were discussing this, it occurred to me that my friend Dr. C. Armstrong, of Carrollton, Illinois, had an aspirator, and he was telegraphed for. He arrived in company with Dr. E. B. Hobson, of Carrollton, Illinois, at 5 P. M The diagnosis of rupture of the urethra was confirmed, and the needle of the aspirator was at once introduced through the abdominal parietes into the bladder. Thirty-six ounces of dark urine were drawn off through the tube, and the patient at once experienced great relief. No anæsthetic was given, but no pain was complained of. No anodyne was administered after the operation, but the patient soon fell into a quiet sleep. The ice bag to the perinæum was continued.

22*d*, 9 A. M.—Patient in distress from distention of the bladder, and begged me to use the aspirator again, but, fearing to do so too frequently, I refused. The introduction of a No. 8 catheter was attempted, but without success, and he was stimulated with brandy and beef tea, and exhorted to exercise patience until afternoon. At 4 P. M., with the assistance of Dr. Fenity, thirty-two ounces of urine were again drawn by the use of the aspirator, immediate relief being experienced. An anodyne was administered.

23*d*, 8 A. M.—Much the same as on the day before, and at 4½ P. M. the aspirator tube was again introduced, and thirty-four ounces of urine withdrawn. Dr. A. B. Allen, of Kane, Illinois, was present, and assisted. The ice bag was continued.

24*th*, 9 A. M.—The gentle introuction of the catheter was attempted without success, and in the afternoon thirty-two ounces of urine were again drawn by means of the aspirator. An anodyne was given at night.

25*th*, 9 A. M.—Catheterization was not attempted, and thirty ounces of urine were drawn by aspiration at 5 P. M. The ice bag to the perinæum was discontinued.

26*th*, 9 A. M.—A few drops of urine had passed the natural channels, and by very gentle efforts a No. 7 catheter was introduced into the bladder. From this time the urine was drawn twice daily by means of the catheter, until August 28th, when the patient passed the urine naturally and without difficulty. He made a rapid recovery, and was, when last heard from, quite well. No peritonitis was threatened at any time, but an anodyne was administered each night, except the first, for its prevention.—*N. Y. Medical Journal.*

